# The effect of fine service on customer loyalty in rural homestays: The mediating role of customer emotion

**DOI:** 10.3389/fpsyg.2022.964522

**Published:** 2022-07-26

**Authors:** Bo Xing, Shihan Li, Dingding Xie

**Affiliations:** School of Management, Tianjin University of Commerce, Tianjin, China

**Keywords:** fine service, rural homestay, customer emotion, customer loyalty, mediation role

## Abstract

Rural homestay is an important driver for developing rural tourism, which still grows against the wind in the post-epidemic era of the COVID-19 virus and shows unique attributes that are different from those of the traditional hospitality industry. Based on the five-dimensional model of fine service theory, this study introduces culture as a unique dimension to construct a six-dimensional model of rural homestay fine service and explores the influencing mechanism of rural homestay fine service on customer loyalty. This study successively used expert interviews and questionnaires to develop the structural equation model through SPSS 26.0 and AMOS 24.0. The results showed that culture, as a unique attribute of rural homestay, is another important factor influencing the level of rural homestay fine service besides privacy, responsiveness, empathy, comfort, and psychological quality. Customer emotion of rural homestays significantly impacts customer loyalty and fully mediates the relationship between fine service and customer loyalty. This study verifies the effectiveness of fine service theory in the research of rural homestay good service and provides a new measuring tool, which has the potential to enrich and develop the exploration of fine service.

## Introduction

The outbreak of the COVID-19 virus has made a huge political and economic impact on countries around the world, which also greatly affected the development of global tourism (Han et al., 2021). In the face of the complex international environment, the Fifth Plenary Session of the 19th CPC Central Committee proposed to accelerate the construction of a new development pattern with a focus on domestic economic circulation complemented by an open dual circulation of domestic and global markets promoting each other (Zeng, 2021). The conference pointed out a new direction for the future development of the Chinese tourism industry, i.e., to take the domestic tourism market as the main target and infuse it with new developmental momentum. In the future, the Chinese tourism industry is likely to achieve high-quality development due to the redirected focus on developing the domestic tourism market which will also support and drive the development of the international tourism market. Against the background of the preventive and control measures for the COVID-19 virus, the outbound and long-distance tourism markets were significantly reduced and strictly limited. But in contrast, short-distance tourism, weekend tourism, and urban peripheral tourism were favored by tourists, and consequently, rural tourism has gradually become an appropriate choice. At the same time, the full implementation of the rural revitalization strategy has created huge development opportunities for rural tourism. According to the data released by the Ministry of Culture and Tourism, 515 million domestic tourism trips were made during the National Day holiday in November 2021, recovering to 70.1% of the same period before the COVID-19 pandemic by comparable caliber and achieving a domestic tourism revenue of 389.061 billion yuan.^1^ More and more tourists chose to go to the mountains and fields for quiet time and leisure, to experience the relaxed lifestyle of the countryside. Hiking, flower appreciation, flower/fruit picking, barbecue, fishing, and other experiential tourism projects have become significantly popular, and rural tourism has become very popular.

The development of rural tourism directly drives the increase in demand for rural accommodation. The influx of tourists at different consumption levels has certainly promoted the transformation and upgrading of the rural accommodation facilities and service quality. Due to the high frequency and single destination characteristics of rural tourism, living in different rural homestays has become an inevitable choice for tourists to get a unique and customized experience when they repeatedly come to the same destination. Some rural homestays find that even when they provide good service, they still find it difficult to attract tourists to revisit. This suggests a prominent and increasing gap between the limited standardized service provided by rural homestays and the customized experience expected by the tourists. Rural homestays urgently need to transform and upgrade from their traditional service style to the latest service model which can provide a new balance between standardized services and customized experience.

While providing tourists with a comfortable accommodation and dining environment, rural homestays also shoulder the important responsibility of allowing tourists to immerse themselves in the local culture of rural characteristics and farming. For rural homestays, farming and agricultural culture are important sources to create unique and customized living experiences for tourists. For example, some rural homestays provide their customers with picking (harvesting) different agriculture products in different seasons or invite them to participate in local special festivals such as the Harvests Festival. Tourists who visit these homestays can still have different living experiences each time, even if they were staying in the same rural homestay.

Against this background of increasing demand for rural accommodation, particularly modern and upgraded accommodation, what kind of rural homestay service can be considered a good service? What kind of service can make tourists feel happy and amazed, and their stay unforgettable? And as an important driver to providing a customized tourism experience in a single tourist destination, will good rural homestay service be able to attract customers to revisit? If the answer is no, what can rural homestays do to attract tourists to visit again?

To answer the above questions, this study takes rural homestay as the research object and fine service as the basic theory to explore the relationship between good service and customer loyalty to rural homestay. As a theoretical concept of good service and high-quality service, fine service aims to get the best customer value by finding an appropriate balance between standardized service and customized experience, that can also meet customers' expectations on privacy, responsiveness, empathy, comfort, and psychological quality (Xing and Bai, 2014). In addition to the above five factors, the study introduced culture as the sixth factor into the fine service model to reflect the characteristics of rural homestays better, given that farming and agricultural culture are important sources for customized living experiences in rural homestays. After developing the six-dimensional scale of rural homestay fine service, the study explored the relationship and mechanisms between fine service and customer loyalty to rural homestays by constructing a structural equation modeling (SEM). Finally, the study developed a new model and a new scale which included culture along with the other five factors to measure the fine service level of rural homestays and found that customer emotion served as a full mediator between fine service and customer loyalty in rural homestays. The conclusion of this study does not only enrich and develop fine service theory but also provides some useful tips for rural homestays to improve their service and attract more customers to come back.

## Literature review and research hypotheses

### Rural homestay

Rural homestay is an important branch of homestay. The fast-paced and high pressure of urban life have led urbanites to yearn for leisurely country life, trying to find a paradise for a short break during their busy lives, while the development of rural tourism has provided a place for urbanites to “escape” from the city, and rural homestay has gradually come into public focus. Gunasekaran and Anandkumar (2012) found that family atmosphere, value for money, contact with local people, and host-guest relationship were important factors influencing tourists' choice of homestay. Zhang (2019) found that tourists paid the most attention to the facilities available in the rooms, geographical location, and service attitude of the homestay. Qiao et al. (2021) analyzed the number of homestays among high-end rural homestays through a geographically weighted regression method to analyze the influence of external environment and internal characteristics on the prices of high-end rural homestays. Zhang et al. (2020) considered rural homestay as an emerging business model and spatial utilization emerging in the process of rural revitalization and tourism transformation and upgrading in China. They flagged that it was also an important business model for rural tourism development and an important opportunity to achieve rural revitalization (Wang and Zeng, 2021).

### Fine service

Fine service is a theoretical concept about various good service-related concepts such as excellent service and high-quality service. After long-term practical observation and study of the service industry, some scholars found that value creation in service interactions has become significantly important. They advocated the integration of customer emotional value with service improvement to create a new theoretical concept called fine service and defined it as a kind of service model with customer emotional value at its core and personalized experience as the main form (Li, 2010). Bai et al. (2010) constructed a service spectrum to explain the different kinds of good service in practice, and placed standard service and fine service at two opposite endpoints. They pointed out that every service enterprise could make a service position in the service spectrum, which could help them to improve their service. Wang and Wang (2014) continued to study the connotation of fine service by using case studies and pointed out that fine service refers to the theory of providing customized services and creating emotional value to meet customers' personalized needs from the customers' perspective, and continuously optimizing the service system and integrating service resources through organizational support, organizational construction based on service assurance, and humanization of the service interface. The enterprise which provides fine service will optimize service systems, integrate service resources, improve service processes, and ultimately achieve continuous improvement of service quality.

Through a content analysis of customer evaluations, Xing and Bai (2014) constructed a theoretical framework of fine service from the perspective of customers through the grounded theory research method of multiple cases. They pointed out that fine service was a conceptual model about the phenomenon of every kind of good service. It was constrained by service productivity and relied on the organization's culture, brand building, and staff support to get the largest customer value by creating an ideal ratio between standardized service and fine experience, which can meet customers' value need for privacy, responsiveness, empathy, comfort, and psychological quality.

For decades, fine service theory has been used to explore the research questions related to tourist destinations resulting in significant achievements. Bai and Liu (2019) carried out multiple case studies of tourist destinations in China and abroad through the grounded theory to explore the connotation of tourist destination fine service. They indicated that the tourist destination fine service was a series of comprehensive services with the core of improving the emotional value of tourists and called for coordination between the local government at the tourist destination, the tourism enterprises, and local residents. They also believed that this kind of service features universal benefit, credibility, goodwill, accessibility, and freedom of choice. They flagged five ways to implement fine service in tourist destinations, including direct supply path of local government, collaborative supply path of government and enterprises, collaborative supply path of government and citizens, self-conscious supply path of tourism enterprises, and self-conscious supply path of residents.

Wei et al. (2021) explored destination fine service with cross-organization and multi-subject participation. Using comparative analysis, they identified three types of organizational relationships and three key driving factors of organizational relationship evolution in the provision of destination fine service. It included the relationship types of fusion, conduction, and coupling, and key driving factors of management flexibility, technology intake, and cultural fit, all of which interact and support each other.

### Fine service and culture

In addition to the five universal factors in fine service theory (privacy, responsiveness, empathy, comfort, and psychological quality), different service industries require the inclusion of additional unique factors according to their industry characteristics. For example, many studies have pointed out that culture is one of the important features of homestay, which is different from the traditional hotel (Huang and Bing, 2021). This applies equally to rural homestays.

In recent years, farming and agricultural cultures as basic unique cultural resources of rural tourism have gradually become a top priority in the rural tourism development process. Promoting farming culture and experiencing farming life has become the core attraction of rural tourism and have become the drivers promoting rural tourism and rural homestay development. He and Zhao (2022) found that the most intuitive representation of rural homestay cluster development was the rural physical landscape, while the rural culture was the essence. Liu et al. (2021) believed that rural homestay was the carrier of historical culture and real life. Integrating cultural connotation into the process of shaping the environment in rural homestays can not only give a new impetus to the environmental space but also make people feel relaxed and comfortable.

In addition, the culture of rural homestays also has an important impact on tourist perception. Sun et al. (2020) carried out a tourist survey based on the cognitive-emotional-intentional relationship theory and found that the homestay which was integrating local folk customs and cultural creativity can provide tourists with distinctive emotional service value. Jiao et al. (2017) found that tourists' perception of the authenticity of local culture created a positive change in tourists' emotions and enhanced particular feelings such as appreciation and admiration. Chen and Xu (2021), through an empirical analysis, proved that including folk customs and rural culture had a significantly positive impact on the emotion and loyalty of tourists. Thus, this study proposes the following hypothesis.

**Hypothesis 1:** the culture of rural homestay is another important dimension of rural homestay fine service besides privacy, responsiveness, empathy, comfort, and psychological quality.

### Fine service and customer emotion

Customer emotion, as a subfield of emotion theory research, first originated from a psychological perspective (Wang and Du, 2013). As competition intensifies in the service industry, customers begin to focus on service enterprises that can give them a pleasant experience. This has increased the attention on customer emotion.

The important difference between fine service theory and service quality theory is that fine service theory places customer emotion at the core of the service (Bai et al., 2010; Li, 2010; Xing and Bai, 2014). Many studies have confirmed that good service creates positive customer emotion, while bad service generates negative customer emotion. Bitner et al. (1990) found that if the service providers couldn't meet customers' standards or expectations, customers feel bad and negative. Oliver (1997) pointed out that excellent service could make customers feel good and even excited. Some scholars explored the relationship between fine service and customer emotion. Li (2010) constructed a conceptual framework for fine service, which was based on customer emotional value, and the study also found that creating memorable experiences for the customers could enhance the emotional connection between customers and enterprises. Wang et al. (2013) proposed the concept of the service spectrum, revealing that customer emotion plays an important role in the process of service interaction. They also found that fine service could create a more positive service experience for customers. Xing and Bai (2014) found that the enterprises with a higher level of fine service always had higher customer emotional levels in the customer service process. Elisabeth et al. (2020) found that the sensory experience of tourists had a positive impact on their emotions in rural environments. Thus, this study proposes the following hypothesis.

**Hypothesis 2:** the fine service level of rural homestays has a positive impact on customer emotions.

### Fine service and customer loyalty

Oliver (1999) argued that customer loyalty is the deep commitment customers have to a preferred product or service, which will lead to repeat purchases of the same brand or the same product. Beyond repeat purchases, inertia thinking meaningfully and positively enhances consumer loyalty (Shi et al., 2018).

Xing and Bai (2014) verified the positive relationship between fine service and customer loyalty by constructing a structural equation model. As the theoretical conception of good service and high-quality service, the conclusion about fine service can also be seen in many studies on the relationship between service quality and customer loyalty. Rust et al. (1995) suggested that enterprises could improve customer loyalty by improving the service quality. Chayuth et al. (2015) confirmed that perceived service quality significantly affected customer emotion and customer satisfaction, which together impacted customer loyalty. Priporas et al. (2017) found a positive correlation between service quality and customer loyalty in Airbnb through empirical research.

Zhang et al. (2017) pointed out that rural tourism's local characteristics could enhance the tourists' local attachment and improve tourists' loyalty. Lu and Fu (2018) took the red tourist attractions as an example and verified that the higher the quality of tourism service, the higher the number of tourist revisits. Lv et al. (2021) found a positive relationship between service attitude and customer sensory experience and loyalty based on online reviews. Han et al. (2021) also verified that the public health service quality of tourism had a significant positive impact on tourist loyalty from the perspective of public health services. In addition, socially sustainable practices and environmentally sustainable practices of hotels can positively impact customer loyalty (Zhou, 2022). Thus, this study proposes the following hypothesis.

**Hypothesis 3:** the fine service level of rural homestays has a positive impact on customer loyalty.

### Customer emotion and customer loyalty

Research on customer loyalty is often inseparable from some variables such as customer satisfaction and customer emotion. Having an appropriate and advanced analytical framework is essential to measuring customer satisfaction (Lin and Vlachos, 2018). Similarly, a correct assessment of the role of customer emotion is required for improving customer loyalty. Oliver (1997) identified three dimensions of customer loyalty—cognition, emotion, and behavioral intention. In the context of the hospitality industry, customer emotion is considered an important prerequisite of customer loyalty. Jonathan and Leonard (2002) highlighted that positive emotions induced customer loyalty, and luxury hotels that can evoke these emotions receive a higher level of brand loyalty. By investigating the relationship between service experience, customer emotion, satisfaction, and price in Chinese resorts, Ali et al. (2015) found a significant relationship between service experience and customer emotion, which jointly influenced customer satisfaction. Beomjoon and Hyun (2020) found that customer emotion had a significant positive impact on customer loyalty when exploring the relationship between customer interaction quality and customer attitude loyalty. Liu (2020) concluded that customer loyalty was positively influenced by customer satisfaction and relationship trust. Jia and Wei (2022) found that the customer's perceived value had a positive effect on customer loyalty in the retailing industry, and the customer's emotional perceived value was the most important influencing factor.

Lv et al. (2020) found that positive sensory impressions have a positive effect on loyalty while negative sensory impressions have a negative effect. Positive customer emotions increase life satisfaction, and life satisfaction impacts the value co-creation between customers and homestays (Lin et al., 2017), which can shape customer loyalty. Chen and Xu (2021) found that customer emotion was the main factor defining customer loyalty in rural homestays through empirical analysis. Thus, this study proposes the following hypothesis.

**Hypothesis 4:** customer emotion toward rural homestay has a positive impact on customer loyalty.

**Figure 1:**
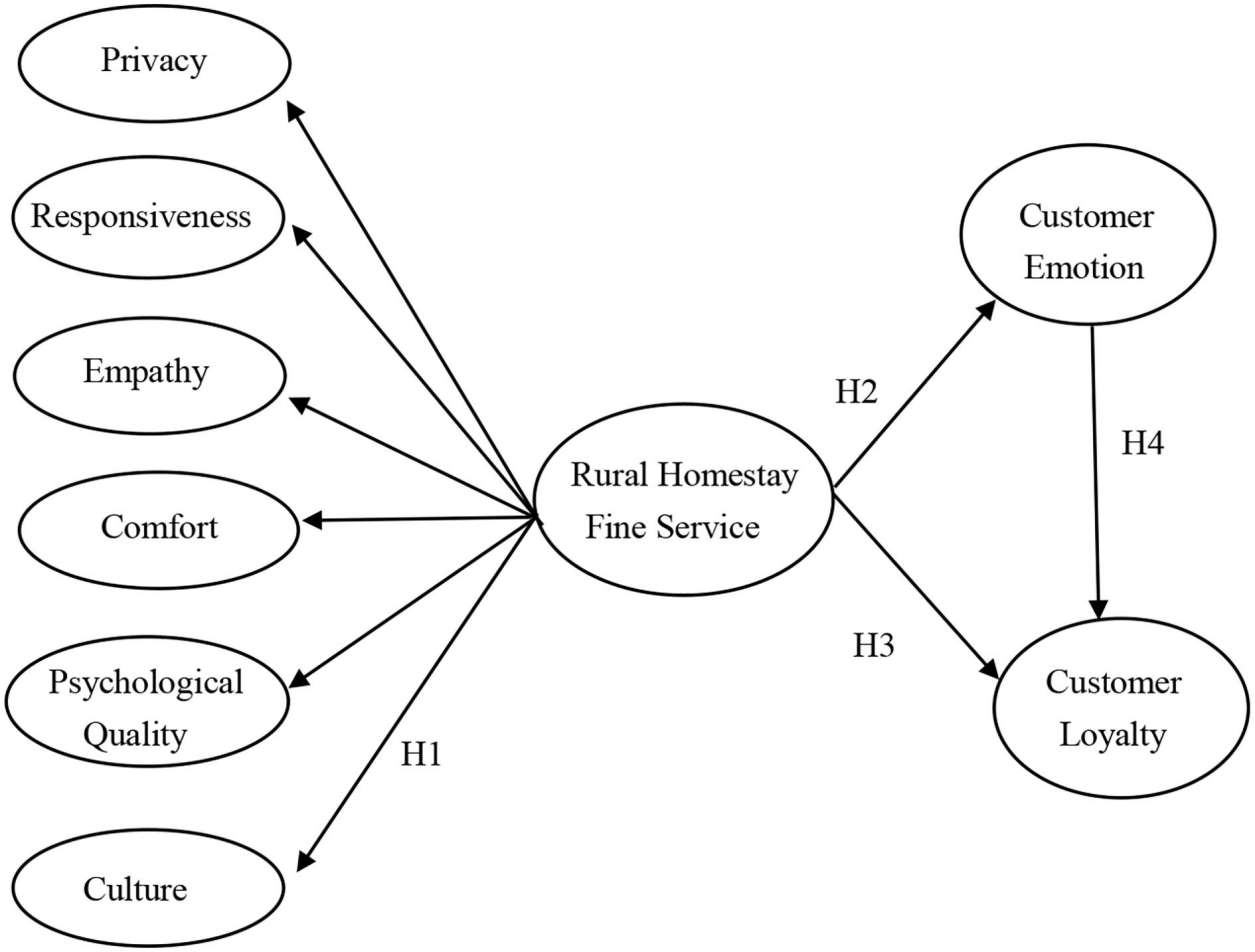
Research model.

The research model is shown in Figure 1.

## Methodology

### Step one: Customizing fine service model for rural homestay

#### Measurement scales and questionnaire design

For the measurement of universal influencing factors of rural homestay fine service, this study used the fine service scale (FS scale) which was proposed and validated by Xing and Bai (2014) as the basis. The FS scale uses privacy, responsiveness, empathy, comfort, and psychological quality as independent variables, and each item has three independent measurement questions. In our H1, culture plays an important role in rural homestays and is believed to be the main dimension influencing the level of fine service. In order to verify the applicability of the fine service scale (FS) in the field of rural homestay, to test the influence of culture on rural homestay fine service, and to construct an observational model, the study adopted the Delphi expert interview method and invited 21 experts and scholars from 12 universities and tourism service enterprises, including Peking University, Nankai University, and Sun Yat-sen University, to give their opinions and suggestions on the dimensions of rural homestay fine service (Table 1).

**Table 1:** Expert information.

**Characteristics**	**%**
**Gender**	
Male	47.6
Female	52.4
**Workplace**	
985 Project	33.3
211 Project	19
Regional key universities	42.8
Tourism enterprise managers	4.7
**Professional title**	
Lecturer	42.8
Associate professor	33.3
Professor	23.8
**Research field**	
Tourism and hotel management	57
Enterprise and service management	33
Culture and cultural economy	10

In the first round of consultation, almost all experts thought the original five dimensions of fine service were suitable for studying rural homestay fine service, while the expressions of some items needed to be adjusted. On the new dimension of culture, more than half of the experts stated that the rural homestay fine service should consider the influence of rural cultural factors and shared many useful suggestions such as “rural homestay needs to provide catering services which can present the local culture,” “rural homestay fine service needs to consider the integration of destination culture and needs to show the respect on the culture of tourist source areas,” and “ the rural homestay culture should reflect local characteristics.” To summarize, they suggested that the cultural characteristics of rural homestays should focus on and reflect regional characteristics. The feedback from the expert consultation was shared with other experts for further consultation to get unanimous approval. As a result of these consultations, this study introduced culture into the FS scale to construct a new scale with six dimensions. Adjustments and optimizations were carried out based on the opinions and suggestions of experts and scholars, and eventually developed the measurement scale of rural homestay fine service.

The indicators for privacy, responsiveness, empathy, comfort, and psychological quality of fine service were derived from Xing and Bai (2014), and the indicators for culture were derived from the expert interviews. For measurement, participants responded using a 5-point Likert scale ranging from 1 (strongly agree) to 5 (strongly disagree). After revising and modifying the initial questions, we undertook a pre-survey on a small scale with 150 participants to test the reliability and validity of each dimension. The results showed that the Cronbach's alpha of each dimension was between 0.707 and 0.864, and the KMO was between 0.670 and 0.723. Among the six dimensions, the Cronbach's alpha and KMO for culture were 0.787 and 0.670, which indicated the scale for each dimension was suitable for measuring. Finally, the study prepared the formal questionnaire in step one, which included six dimensions and 18 indicators (Table 2).

**Table 2:** Measurement scales of step one.

**Independent variables**	**Measurement questions**
Privacy	AA1.The service space of X homestay has good privacy.
	AA2. X Homestay strictly keeps customer information confidential.
	AA3. X homestay respects customer privacy and does not disturb easily without need.
Responsiveness	BB1. X homestay always respond quickly when I make a request to the homestay.
	BB2. When there is a problem with the service, X homestay is always deal with the problem as soon as possible.
	BB3. When I am in trouble, the service staff of X homestay is always willing to help me.
Empathy	CC1. It is pleasant to communicate with the service staff of X homestay.
	CC2. The service staff of X homestay always take the initiative to care about the customers.
	CC3. X homestay service staff always can provide reasonable suggestions for me.
Comfort	DD1.The architecture and decoration style of X homestay is exquisite and individual.
	DD2.The room of X homestay makes me feel very comfortable.
	DD3. The supporting facilities of X homestay are attractive to me.
Psychological quality	EE1. X homestay has a good reputation in the industry.
	EE2. Other customers staying at X homestay are similar to me in some ways.
	EE3. Staying at X homestay is a symbol of identity.
Culture	FF1. The staff of X homestay is familiar with the local culture and customs.
	FF2. The facilities and decoration of X homestay have a distinctive regional character.
	FF3. X homestay offers cultural activities which can present local characteristics.

### Step two: Mediating effect on customer emotion

#### Measurement scales and questionnaire design

The indicators for customer emotion were derived from Anderson and Anderson and Weitz (1992) and Ganesan (1994). The indicators for customer loyalty were derived from Parasuraman and Zeithaml (1994); Barnes (1997); Bettencourt (1997); Cronin et al. (2000), and Kennedy et al. (2001). For measurement, participants responded using a 5-point Likert scale ranging from 1 (strongly agree) to 5 (strongly disagree). After a pre-survey on a small-scale and result analysis, the formal questionnaire was prepared, which included five indicators for each variable (Table 3).

**Table 3:** Measurement scales of step two.

**Concepts to be tested**	**Measurement questions**	**Sources**
Customer emotion	CE1. I like X homestay very much. (a) (b)	(a) Anderson and Weitz, 1992
	CE2. I have a special love for X homestay. (a) (b)	(b) Ganesan, 1994
	CE3. I am very concerned about the service quality improvement of X homestay. (a) CE4. I'll tell the person around me that I like/love X homestay. (b) CE5. Choosing X homestay makes me feel security. (a)	
Customer loyalty	CL1. I'll say good words about the X homestay after I am leaving. (a) CL2. I will book X homestay again. (b) CL3. When I need to book a homestay, I always choose X homestay (c). CL4. I'd like to provide advice to help X homestay improve its service quality. (d) CL5. The reason why I choose X homestay is that it really makes me satisfied, not because I have no choice. (e)	(a) Parasuraman and Zeithaml, 1994 (b) Kennedy et al., 2001 (c) Bettencourt, 1997 (d) Cronin et al., 2000 (e) Barnes, 1997

### Data collection

The study collected research data through two channels. Firstly, a convenience sampling method was used to distribute questionnaires through online social media platforms, through which we collected 162 questionnaires. And then a representative sampling method was used to distribute questionnaires in the rural tourist attractions, through which we received 156 questionnaires. After excluding 25 invalid questionnaires, 293 valid questionnaires were eventually selected, and the questionnaire effective rate was 92.1%.

## Results

### Descriptive statistics

As shown in Table 4, 28.7% of the respondents (*n* = 84) were male and 71.3% (*n* = 209) were female. 85.7% of them (*n* = 251) were between 18 and 30 years of age, and 9.6% (*n* = 28) were between 36 and 45 years of age. On the level of education, 62.1% (*n* = 182) of the respondents had a bachelor's degree and 34.5% (*n* = 101) had a master's degree and above.

**Table 4:** Sample profile (*n* = 293).

**Characteristics**	**N**	**%**
**Gender**		
Male	84	28.7
Female	209	71.3
**Age**		
≤24	139	47.4
25–30	112	38.2
31–35	9	3.1
36–45	28	9.6
46–60	5	1.7
**Education level**		
High school and below	1	0.3
Junior college	9	3.1
Undergraduate	182	62.1
Master's degree and above	101	34.5
**Annual household income**		
≤50,000yuan	111	37.9
50,000–100,000 yuan	71	24.2
110,000–200,000 yuan	60	20.5
200,000–300,000 yuan	27	9.2
>300,000 yuan	24	8.2
**Number of homestay experience**		
1–2	110	37.5
3–4	72	24.6
≥5	111	37.9
**Tourist type**		
Personal independent travel	46	15.7
Couples/friends independent travel	193	65.9
Parent-child travel	36	12.3
Multiple family independent travel	14	4.8
Group tour	4	1.4

In the aspect of annual household income, 37.9% of the respondents (*n* = 111) made <50,000 yuan a year and 24.2% (*n* = 71) made 50,000–100,000 yuan a year. This is because, in the past, rural tourism was regarded as poor man's travel and rural homestay was also regarded as a kind of budget accommodation comprising mostly of farmhouses. In recent years, especially after the outbreak of the COVID-19 virus, more and more high-income group tourists have chosen rural tourism as a substitute for long-distance or outbound tourism. The income level of the rural homestay's target market has, therefore, increased accordingly. In this study, 29.7% of the respondents (*n* = 87) made 100,000–300,000 yuan a year and 8.2% (*n* = 24), more than 300,000 yuan a year.

The higher the annual income level of the tourist, the higher the frequency of rural tourism and living in rural homestays. In this study, 62.5% of the respondents (*n* = 183) had stayed at rural accommodations more than three times, of which 37.9% (*n* = 111) had been on rural homestays more than five times.

On tourist types, 65.9% of the respondents (*n* = 193) traveled with their lovers or friends, and 12.3% (*n* = 36) traveled with their family, especially their kids. Table 4 summarizes the respondents' details.

### Exploratory factor analysis and reliability test

To verify the research hypotheses, the study first used Cronbach's alpha to test the internal consistency of each factor and each variable using SPSS26.0. The results showed that the Cronbach's alpha of each factor and each variable was between 0.870 and 0.940, which exceeded the threshold of 0.70, and the deletion of each question significantly reduced the reliability of the sub-scale. This result indicated that the overall reliability of the scale and each factor and variable was very good and is suitable for further analysis.

Secondly, the study used EFA to measure the rural homestay fine service scale which included six factors, and used principal components factor analysis and varimax rotation to perform factor extraction. The KMO and Bartlett test can verify the appropriateness of factor analysis in the data. After calculation by SPSS26.0, the KMO measure of 0.940 showed that the factor analysis was suitable. From another perspective, the Bartlett test of sphericity of 0.00 also indicated that the variables were suitable for factor analysis.

The statistical result extracted six common factors where the items of each were consistent with the questions contained in each subscale, and the factor loads of each item were higher than 0.5, and the explained variance percentage reached 78.46%. In summary, the six-dimensional model structure of the rural homestay fine service was preliminarily verified (Table 5).

**Table 5:** Factor loadings for each item of the FS scale.

	**A Privacy**	**B Responsiveness**	**C Empathy**	**D Comfort**	**E Psychological quality**	**F Culture**
A1	0.803					
A2	0.802					
A3	0.605					
B1		0.763				
B2		0.724				
B3		0.655				
C1			0.798			
C2			0.643			
C3			0.618			
D1				0.680		
D2				0.669		
D3				0.646		
E1					0.800	
E2					0.609	
E3					0.502	
F1						0.764
F2						0.760
F3						0.564

### Confirmatory factor analysis

To verify hypothesis one, the study compared the five-dimensional model of fine service with the six-dimensional model after adding culture. The fitting parameters and confirmatory factor analysis were performed using AMOS24.0, and the results showed that all the final CFA models had good fitting index. Overall, the fitting index of the six-dimensional model (CMIN/DF = 2.692, RMSEA = 0.076, NFI = 0.889, CFI = 0.926, PNFI = 0.686, PCFI = 0.714) was better than that of the five-dimensional model (CMIN/DF = 2.810, RMSEA = 0.079, NFI = 0.905, CFI = 0.936, PNFI = 0.681, PCFI = 0.704).

As shown in Table 6, the CR values of all six dimensions ranged from 0.678 to 0.848, and the average variance extracted (AVE) values were from 0.421 to 0.651 with most of them >0.5. This result showed that the scale had good convergent validity.

**Table 6:** Convergent validity.

**Latent variable**	**Observation variable**	**Std**.	**Unstd**.	**S.E**.	**C.R**.	**P**	**SMC**	**CR**	**AVE**
	A1	0.783	1				0.613	0.4	0.595
Privacy	A2	0.834	0.962	0.071	13.523	000	0.696	0.814	0.595
	A3	0.69	0.731	0.066	11.152	000	0.476		
	B1	0.835	1			0.697		
Responsiveness	B2	0.85	1.041	0.064	16.378	000	0.723	0.848	0.651
	B3	0.73	0.848	0.065	13.044	000	0.533		
	C1	0.647	1				0.419	0.5	0.551
Empathy	C2	0.807	1.635	0.154	10.607	000	0.651	0.785	0.551
	C3	0.764	1.409	0.137	10.284	000	0.584		
	D1	0.719	1				0.517	0.0	0.620
Comfort	D2	0.825	1.029	0.081	12.666	000	0.681	0.830	0.620
	D3	0.813	1.134	0.09	12.58	000	0.661		
	E1	0.8	1				0.640	0.8	0.421
Psychological quality	E2	0.609	0.745	0.076	9.807	000	0.371	0.678	0.421
	E3	0.502	0.794	0.1	7.942	000	0.252		
	F1	0.589	1				0.347		
Culture	F2	0.834	1.604	0.169	9.479	000	0.696	0.782	0.550
	F3	0.78	1.41	0.153	9.241	000	0.608		

In Table 7, the square root value of AVE is greater than the correlation coefficient between each dimension, indicating that each dimension had a high discriminative validity (Majeed et al., 2020).

**Table 7:** Discriminative validity.

	**AVE**	**Culture**	**P Q**	**Comfort**	**Empathy**	**Responsiveness**	**Privacy**
Culture	0.550	0.742					
P Q	0.421	0.214	0.649				
Comfort	0.620	0.204	0.320	0.787			
Empathy	0.551	0.150	0.235	0.224	0.742		
Responsiveness	0.651	0.222	0.347	0.331	0.243	0.807	
Privacy	0.595	0.194	0.305	0.290	0.213	0.315	0.771

All the above data analyses indicate that compared with the five-dimensional model of fine service, the six-dimensional model with culture included reflects a more comprehensive display of rural homestay fine service. So, hypothesis one was verified.

### Mediating effect on customer emotion

**Figure 2:**
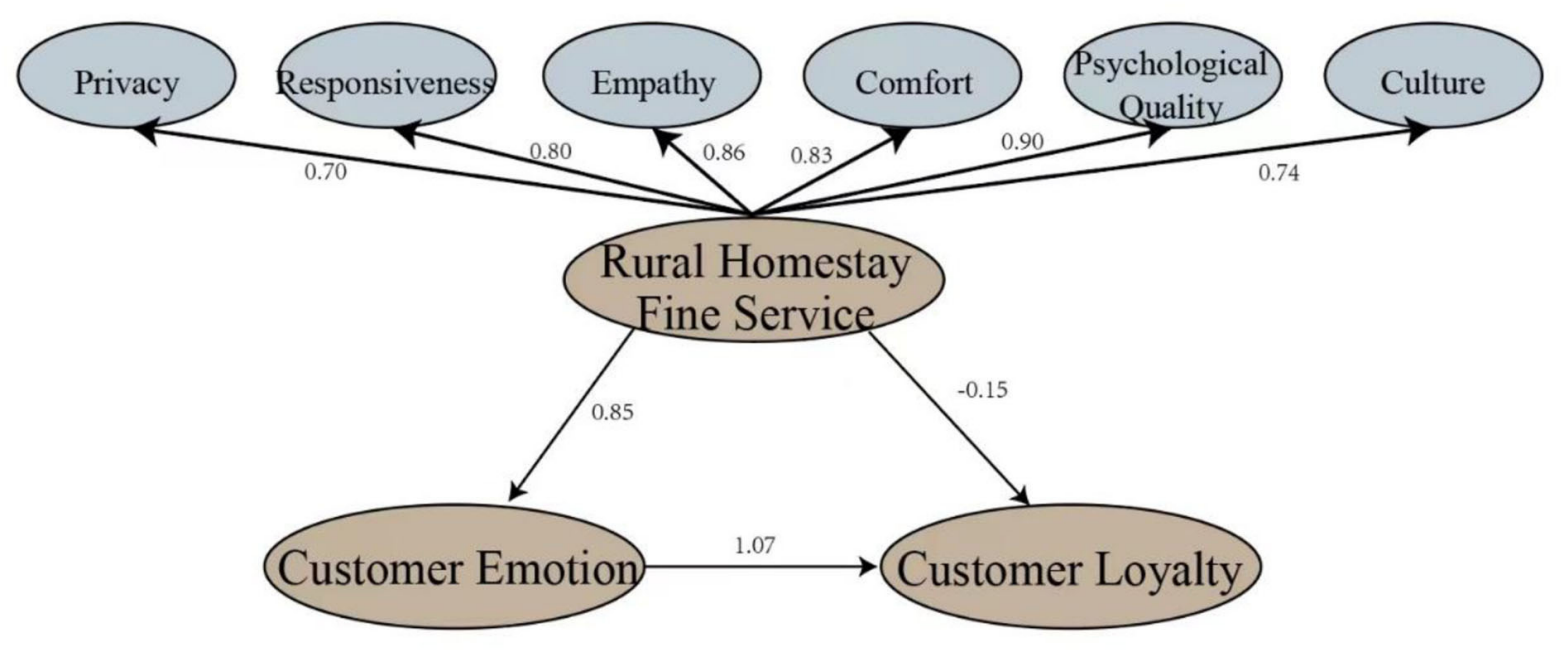
Results of SEM analysis.

To test the relationship between rural homestay fine service, customer emotion and customer loyalty, and verify the mediating effect of customer emotion, the study fit the hypothesis model using the SEM method (Figure 1). After the calculation by AMOS 24.0, as shown in Figure 2, all six dimensions showed positive effect on fine service (Table 8), and fitting indexes were accepted (CMIN/DF = 2.745, RMSEA = 0.077, NFI = 0.821, CFI = 0.878, PNFI = 0.741, PCFI = 0.792). This indicated that the hypothetical model had good internal quality.

**Table 8:** Results of path verification.

	**Std**.	**Unstd**.	**S.E**.	**C.R**.	**P**	**Test results**
Privacy → FS	0.701	0.907	0.098	9.297	000	–
Responsiveness → FS	0.797	1.035	0.097	10.703	000	–
Empathy → FS	0.862	0.701	0.078	8.943	000	–
Comfort → FS	0.825	0.953	0.096	9.973	000	–
Psychological Quality → FS	0.903	1.102	0.119	9.297	000	–
Culture → FS	0.741	0.638	0.083	7.65	000	H1: support
FS → CE	0.854	1.052	0.093	11.332	000	H2: support
CE → CL	1.07	1.077	0.132	8.152	000	H4: support
FS → CL	−0.152	−0.189	0.14	−1.353	0.176	H3: reject

In terms of the relationship between rural homestay fine service, customer emotion, and customer loyalty, Table 8 shows that the direct impact of fine service on customer loyalty is not obvious (*p* = 0.176), so hypothesis three was rejected. On the other hand, the level of rural homestay fine service can significantly affect customer emotion (β = 0.854, *p* < 0.001), and customer emotion also has a significant effect on customer loyalty (β = 1.07, *p* < 0.001), which shows hypotheses two and four were supported. In rural homestays, customers' emotion plays a complete mediating role between fine service and customer loyalty.

## Discussion and conclusion

### Conclusion

Firstly, as an independent and complete theoretical concept to explain different kinds of good service, fine service theory is suitable to explain the good service in rural homestays. Fine service is constrained by service productivity and relies on the organization's culture, brand building, and staff support to receive maximum positive feedback and score from customers. This can be obtained by ensuring an ideal ratio between standardized service and fine experience that can meet customers' values and need for privacy, responsiveness, empathy, comfort, and psychological quality (Xing and Bai, 2014). The empirical results of this research also verified these five factors that influence the level of rural homestay fine service.

Secondly, beyond these five factors, culture is another important influencing factor of rural homestay fine service. As a special resource, exposure to farming and agriculture are priceless treasures of rural homestay and rural tourism. This is the biggest difference between rural homestays and other forms of accommodation. The rural culture can give tourists a unique and memorable living experience, which can attract them to visit and live in rural homestays again and again.

Thirdly, customer emotion plays a complete mediating role between fine service and customer loyalty. This finding is different from similar analyses of other service industries where customer emotion usually plays a partial mediating role. The reason for this conclusion may be the particularity of customers' purchasing behavior in rural homestay. As a type of accommodation, the rural homestay is only a part of the whole rural tourism. To feel fresh on each trip, without changing their destinations, most tourists prefer to change their rural homestays to pursue different experiences, no matter whether the rural homestay service is fine or not. So, the good service of a rural homestay cannot necessarily attract the customer to revisit. But for experienced tourists, a rural homestay that they like or love will get more attention when they are booking or making purchase decisions. It is also easier to obtain positive word-of-mouth advertisements and referrals because emotions are essentially an important part of the rural tourism experience.

### Contributions

Firstly, the study enriches and develops fine service theory by using it to explore the inter-relationship and dynamics between rural homestay service, customer emotion, and customer loyalty. As a theoretical concept of good service and high-quality service, fine service theory is more comprehensive and appropriate in explaining the phenomenon of different kinds of good service because customers' expectations can be fully addressed. Although previous research confirmed the applicability and effectiveness of fine service theory, the adaptability of fine service theory in a specific service sub-industry is still under research. Exploring rural homestay fine service is not only a new perspective to learn about the rural homestay industry but also an application and development research of fine service theory in a specific sub-industry. Fortunately, the results are good, and the fine service theory was verified to be effective in this study on a specific service sub-industry, namely the rural homestay.

Secondly, the study confirms the importance of culture in rural homestay service and has developed a six-dimensional scale to measure the level of fine service in rural homestays. The fine service scale which includes privacy, responsiveness, empathy, comfort, and psychological quality is good for assessing the level of fine service in rural homestays. When culture was added as a factor, the explanatory power of the rural homestay fine service model improved significantly. This result shows that the rural culture including farming and agriculture is a very important influencing factor in rural homestay fine service. And the six-dimensional scale is better suited as a measuring tool.

### Management implications

From the perspective of management practice, the study provided a lot of effective service strategies for rural homestay operation and management. First, rural homestays should dispel the customers' concerns about security by protecting their privacy. As customers increase, they will start paying more attention to personal privacy protection. Second, rural homestays should offer user-friendly accommodations that respond to the customers' needs and helps them by solving problems as soon as possible and making them feel valued. Third, rural homestay should focus on a good service attitude and treat customers as their friends, or even their families, to create a wonderful and warm living experience just like the customers' own home, which can make customers feel relaxed and comfortable. Fourth, rural homestays should pay more attention to interior design and room decoration. A comfortable and characteristic room can give customers the feeling of warmth, pleasant experience, and amazement. Fifth, the customer is also a very important part of the rural homestay image. People often like to live with people who are similar to themselves. If the customers who visit the rural homestay are equally polite and friendly, they will feel very good no matter if they know each other or not. To provide a good experience and a peaceful environment, rural homestays should have clear customer guidelines and set a certain standard to select their customers by parameters such as price. Sixth, since culture is an important influencing factor of rural homestay fine service, some cultural activities that can provide exposure to the local farming and agricultural culture should be designed into the homestay living experience. The immersive cultural experience should increase customers' participation and make them feel engaged, which can create a different living experience for them each time. Finally, the fine service of rural homestay can make customers feel good and satisfied, but it cannot guarantee their return. Building positive emotions with customers can help rural homestay to retain their customer, so just giving the customer the feeling of “good” is not enough; to feel wonderful and happy is more valuable.

### Limitation and future research

Affected by the control measures of the COVID-19 virus, the scope of field visits of this study was limited. The field observation of rural homestay was mostly concentrated in the Beijing-Tianjin-Hebei region; therefore, the study has distinct regional characteristics and the research conclusions are a little oversimplified.

The study has verified the positive effect of the rural homestay fine service on customer loyalty through the complete mediation of customer emotion, but the dynamics between fine service and customer emotion have not been answered in this study, which is an important research subject that needs to be explored in the future. For example, do the six dimensions of rural homestay fine service have the same effect on customer emotion? If not, which one has the most significant influence? Are there any variables that play a moderating role between fine service and customer emotion?

On the other hand, in addition to customer emotion, are there other variables that play a mediating role between rural homestay fine service and customer loyalty is also a question that need to be studied. In the existing research, some variables such as customer trust and customer satisfaction may be related to customer emotion and loyalty. What are their roles between fine service and customer loyalty? Are they mediators just like customer emotion or do they perform other roles? What is the relationship and mechanism between them and customer emotion? All these questions need further research and exploration in the future.

## Data availability statement

The raw data supporting the conclusions of this article will be made available by the authors, without undue reservation.

## Ethics statement

Ethical review and approval was not required for the study on human participants in accordance with the local legislation and institutional requirements. Written informed consent from the patients/participants or patients/participants legal guardian/next of kin was not required to participate in this study in accordance with the national legislation and the institutional requirements.

## Author contributions

All authors listed have made a substantial, direct, and intellectual contribution to the work and approved it for publication.

## Funding

This article was funded by the youth project of Philosophy and Social Sciences, Tianjin Province, China (Grant No. TJGLQN18-012).

## Conflict of interest

The authors declare that the research was conducted in the absence of any commercial or financial relationships that could be construed as a potential conflict of interest.

## Publisher's note

All claims expressed in this article are solely those of the authors and do not necessarily represent those of their affiliated organizations, or those of the publisher, the editors and the reviewers. Any product that may be evaluated in this article, or claim that may be made by its manufacturer, is not guaranteed or endorsed by the publisher.

## Footnotes

^1^Source: Ministry of Culture and Tourism. Culture and tourism market during the National Day holiday of 2021 [EB/OL]. https://www.mct.gov.cn/whzx/whyw/202110/t20211007_928156.htm,2021-10-07.
